# Neuropsychiatric Outcomes Associated with GLP-1 Receptor Agonists in Adults with Overweight or Obesity: A Systematic Review

**DOI:** 10.3390/brainsci16090926

**Published:** 2026-08-31

**Authors:** Carla Lubo, Rosa Bustamante, Laura Quintana, Hernan F. Guillen-Burgos

**Affiliations:** 1 Department of Psychiatry, Universidad Simón Bolívar, Barranquilla 080002, Colombia; rosa.bustamante@unisimon.edu.co (R.B.); laura.quintana@unisimon.edu.co (L.Q.); 2 Center for Clinical and Translational Research, Universidad Simón Bolívar, Barranquilla 080002, Colombia; 3 Department of Psychiatry and Mental Health, Pontificia Universidad Javeriana, Bogotá 110111, Colombia

**Keywords:** GLP-1 receptor agonists, obesity, overweight, depressive symptoms, anxiety symptoms, suicidal ideation, suicide attempts, neuropsychiatric outcomes, semaglutide, liraglutide, tirzepatide, systematic review

## Abstract

**Highlights:**

**What are the main findings?**

- This systematic review found no consistent evidence of an increased risk of depression, anxiety, manic/hypomanic symptoms, or suicidal outcomes in adults with overweight or obesity.
- Randomized clinical trials generally showed no worsening of neuropsychiatric outcomes, whereas observational studies yielded heterogeneous findings, with estimates in both favorable and adverse directions.

**What are the implications of the main findings?**

- The available evidence does not support a consistent adverse neuropsychiatric signal associated with GLP-1 receptor agonist use for overweight or obesity; however, the heterogeneity of findings warrants cautious interpretation and continued clinical monitoring.
- Future prospective studies specifically designed to assess neuropsychiatric outcomes are needed to clarify long-term effects and potential differences among individual GLP-1 receptor agonists.

**Abstract:**

**Background/Objectives:** Glucagon-like peptide-1 receptor agonists (GLP-1 RAs) are increasingly used for the treatment of overweight and obesity because of their established efficacy in achieving clinically meaningful weight loss. However, concerns have emerged regarding their potential association with neuropsychiatric outcomes, including depressive and anxiety symptoms, manic or hypomanic symptoms, and suicidal ideation or behavior. This systematic review aimed to synthesize the available evidence on neuropsychiatric outcomes associated with GLP-1 RA use in adults with overweight or obesity. **Methods:** A systematic search of PubMed/MEDLINE, Scopus, and Web of Science was conducted following PRISMA 2020 guidelines. Eligible studies included randomized controlled trials, longitudinal observational studies, and cross-sectional studies evaluating neuropsychiatric outcomes associated with GLP-1 RAs in adults with overweight or obesity. Risk of bias was assessed using RoB 2, ROBINS-I, and the Joanna Briggs Institute Critical Appraisal Checklist according to study design. Given the clinical and methodological heterogeneity of the included studies, findings were synthesized narratively in accordance with SWiM guidance, and certainty of evidence was assessed at the outcome level using GRADE. **Results:** Fourteen studies were included, comprising four randomized studies, nine longitudinal observational studies, and one analytical cross-sectional study. Randomized evidence showed no consistent worsening of depressive symptoms or imbalance in suicide-related outcomes, whereas observational findings were heterogeneous. For depression, observational HRs ranged from 0.63 to 2.95 across different populations and comparators, while randomized studies showed small or no between-group differences in depressive symptoms. For suicide-related outcomes, observational HRs/aHRs ranged from 0.27 to 2.06, while suicidal ideation or behavior was uncommon in randomized studies and showed no consistent treatment imbalance. Anxiety-related findings were also heterogeneous, and manic or hypomanic symptoms were not systematically assessed. GRADE certainty was low for depressive symptoms and very low for anxiety and suicide-related outcomes. **Conclusions:** Current evidence does not demonstrate a consistent increase in adverse neuropsychiatric outcomes associated with GLP-1 receptor agonists in adults with overweight or obesity. Although some studies reported neutral or potentially beneficial associations, observational findings were heterogeneous, and the certainty of evidence was low or very low across the assessable outcomes. These findings do not establish a class-wide neuropsychiatric safety effect, particularly given differences among individual agents, study populations, and baseline psychiatric characteristics. Further prospective studies specifically designed to assess neuropsychiatric outcomes, including individuals with pre-existing psychiatric disorders, are warranted.

## 1. Introduction

Obesity and overweight represent major global public health challenges because of their association with type 2 diabetes mellitus, cardiovascular disease, reduced quality of life, and increased mortality [1,2]. The prevalence of obesity has increased substantially over recent decades, affecting more than two billion individuals worldwide and imposing a considerable burden on healthcare systems [1,2]. Given the chronic and multifactorial nature of obesity, current management strategies combine lifestyle interventions with pharmacological therapies in selected patients [3]. Among these, glucagon-like peptide-1 receptor agonists (GLP-1 RAs) have emerged as highly effective treatments for obesity. GLP-1 RAs have demonstrated clinically meaningful and sustained weight loss in adults with overweight or obesity, together with improvements in cardiometabolic outcomes, leading to their incorporation into contemporary obesity and T2DM management strategies [4,5].

Beyond their metabolic effects, growing evidence suggests that glucagon-like peptide-1 (GLP-1) signaling plays an important role within the central nervous system. GLP-1 receptors are widely distributed in brain regions involved in appetite regulation, reward processing, emotional regulation, learning, memory, and executive functioning, including the hypothalamus, nucleus tractus solitarius, hippocampus, amygdala, nucleus accumbens, and ventral tegmental area [6,7,8]. Experimental evidence indicates that GLP-1 signaling may influence dopaminergic neurotransmission, neuroinflammatory pathways, neuronal plasticity, and stress-related neuroendocrine responses, providing a biological rationale for potential effects on mood and behavior [6,7,8,9]. This relationship is particularly relevant in obesity, a chronic systemic disease that is frequently accompanied by depression, anxiety, impaired quality of life, and other mental health conditions [10,11]. Consequently, as the clinical use of GLP-1 receptor agonists continues to expand, understanding their potential neuropsychiatric effects has become increasingly important for both metabolic and psychiatric care.

Despite their increasing use, concerns have emerged regarding their potential association with neuropsychiatric adverse events, particularly depression, anxiety, manic/hypomanic symptoms, and suicidal ideation/behavior or self-harm. These concerns were amplified by spontaneous reports from pharmacovigilance databases and prompted regulatory reviews by agencies including the European Medicines Agency (EMA) and the U.S. Food and Drug Administration (FDA) [12,13]. However, the available evidence remains inconsistent. Recent studies have yielded conflicting findings: randomized trials have generally shown no worsening of depressive symptoms or suicidal ideation/behavior, whereas observational studies have reported neutral, potentially favorable, and adverse associations with psychiatric outcomes [14,15,16]. Some studies have also reported improvements in depressive or anxiety symptoms and psychological well-being, although these findings do not establish a therapeutic psychiatric effect [17]. Interpretation of this evidence is complicated by important methodological differences across studies, including confounding by indication in observational designs, variation in study populations and active comparators, the use of standardized symptom scales versus diagnostic codes from electronic health records, differences in the ascertainment of suicidal outcomes, and the exclusion of individuals with major psychiatric disorders from several randomized trials [14,15,16]. These limitations, together with heterogeneity in study design, outcome definitions, follow-up duration, and population characteristics, preclude definitive conclusions about the direction and magnitude of potential neuropsychiatric effects of GLP-1 receptor agonists.

Given the growing use of GLP-1 receptor agonists and the conflicting evidence regarding their potential neuropsychiatric effects, an updated synthesis of the available evidence is warranted. Therefore, this systematic review aimed to critically evaluate the association between GLP-1 receptor agonist treatment and neuropsychiatric outcomes, including depression, anxiety, manic or hypomanic symptoms, and suicidal ideation or behavior, in adults with overweight or obesity.

## 2. Materials and Methods

### 2.1. Study Design and Reporting Standards

This systematic review was designed to evaluate the association between glucagon-like peptide-1 receptor agonist (GLP-1 RA) treatment and neuropsychiatric outcomes in adults with overweight or obesity. The protocol was prospectively registered in the International Prospective Register of Systematic Reviews (PROSPERO; CRD420251246255). The review was reported in accordance with the Preferred Reporting Items for Systematic Reviews and Meta-Analyses (PRISMA) 2020 statement [18]. The completed PRISMA 2020 checklist is provided in Supplementary Table S6. Methodological decisions regarding study selection, data extraction, risk-of-bias assessment, and evidence synthesis followed current recommendations from the Cochrane Handbook for Systematic Reviews of Interventions where applicable [19].

### 2.2. Eligibility Criteria

Eligible studies were selected according to the population, intervention/exposure, comparator, outcomes, and study design framework. The population of interest comprised adults aged ≥18 years with overweight or obesity, defined using recognized clinical or anthropometric criteria (BMI ≥ 25 kg/m^2^), with or without T2DM. Eligible exposures included GLP-1 receptor agonists and the prespecified dual GIP/GLP-1 receptor agonist tirzepatide. Studies were required to assess at least one prespecified neuropsychiatric outcome: depressive symptoms, anxiety symptoms, manic or hypomanic symptoms, suicidal ideation, suicide attempts, or self-harm. Randomized and non-randomized clinical trials and observational studies were eligible. Detailed inclusion and exclusion criteria are presented in Table 1.

### 2.3. Information Sources and Search Strategy

A systematic literature search was conducted in PubMed/MEDLINE, Scopus, and Web of Science to identify studies evaluating neuropsychiatric outcomes associated with glucagon-like peptide-1 receptor agonists (GLP-1 RAs) in adults with overweight or obesity. The final search was conducted on 13 June 2026. Embase was not included because institutional access was unavailable. The search was restricted to articles published within the preceding 10 years and to full-text articles published in English or Spanish. The 10-year publication window was used to focus the review on contemporary evidence reflecting the current clinical use of GLP-1-based therapies, while the language restriction was applied to ensure reliable full-text eligibility assessment and data extraction by the review team.

The search strategy combined controlled vocabulary terms (MeSH, when applicable) and free-text terms related to the population, intervention, and outcomes of interest. Terms describing obesity and overweight included “Obesity” [MeSH], obesity, overweight, adiposity, weight loss, and weight management. Terms related to GLP-1 receptor agonists included “Glucagon-Like Peptide 1 Receptor” [MeSH], GLP-1 receptor agonists, semaglutide, liraglutide, dulaglutide, exenatide, lixisenatide, and tirzepatide. Neuropsychiatric outcomes were identified using terms related to depression, anxiety, suicidal ideation, self-harm, mania, hypomania, bipolar disorder, and broader mental health and psychiatric terms. Search syntax was adapted for each database according to its indexing system, interface, and field tags. The complete database-specific electronic search strategies are provided in Supplementary Table S5.

### 2.4. Study Selection

Reference management and study screening were conducted using Rayyan (https://www.rayyan.ai/, accessed on 16 August 2026) [20]. Records retrieved from PubMed/MEDLINE, Scopus, and Web of Science were exported in compatible formats and imported into the platform. Duplicate records were identified using Rayyan’s automated detection tool and subsequently verified manually.

**Figure 1:**
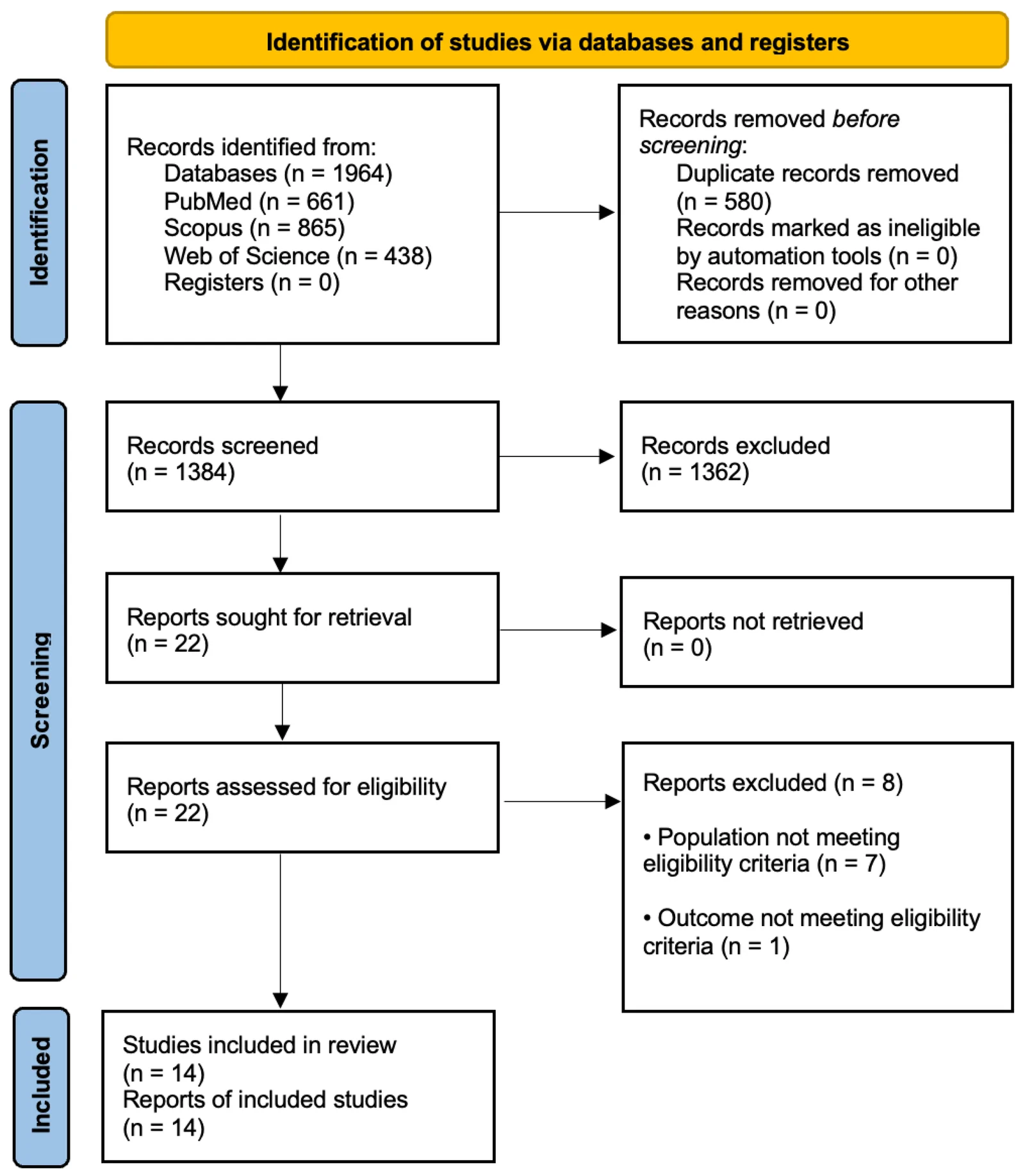
PRISMA 2020 flow diagram illustrating the identification, screening, and selection process of studies included in the systematic review.

Two reviewers (CL, RB) independently screened titles and abstracts, with each reviewer blinded to the other reviewer’s decisions, classifying records as “included,” “excluded,” or “maybe.” Records classified as “included” or “maybe” were retrieved for full-text assessment and evaluated according to the prespecified eligibility criteria. Disagreements were resolved through discussion and consensus and, when necessary, consultation with a third reviewer (HFGB). Reasons for exclusion were documented at the full-text stage. Following peer-review comments, all full-text articles considered for inclusion were reassessed against the prespecified PICO/PECO eligibility criteria, with particular attention to the requirement that study populations comprise adults with overweight or obesity. In addition, studies identified in the original search but not initially included because their full texts had not been retrieved were reassessed after full-text retrieval using the same eligibility criteria. The final set of included studies and the corresponding data extraction were updated accordingly. The study identification, screening, eligibility, and inclusion process is presented in the PRISMA 2020 flow diagram (Figure 1).

### 2.5. Data Extraction

Data extraction was performed using a standardized form. Extracted study characteristics included country, study design, sample size, follow-up duration, and outcome assessment methods. Participant characteristics comprised age, sex, and clinical population. Intervention or exposure characteristics included the type of GLP-1 receptor agonist, dosage, treatment duration, and comparator group when applicable. Outcome data included depressive symptoms, anxiety symptoms, manic or hypomanic symptoms, suicidal ideation, suicide attempts, and self-harm. Information regarding assessment instruments (e.g., PHQ-9, HADS, C-SSRS), diagnostic criteria, effect estimates, confidence intervals, *p*-values, and measures of association reported by the studies was extracted when available. Data extraction and narrative synthesis were conducted by two reviewers (CL and RB) using predefined extraction forms. Extracted information was subsequently reviewed for consistency and methodological accuracy by a third reviewer (HFGB). Any discrepancies in data interpretation or study classification were resolved through discussion and consensus.

### 2.6. Risk of Bias Assessment

Risk of bias was independently assessed by two reviewers (CL, RB) according to study design. Randomized controlled trials were evaluated using the Cochrane Risk of Bias 2 (RoB 2) tool [21]. Non-randomized longitudinal studies were assessed using the Risk of Bias In Non-randomized Studies of Interventions (ROBINS-I) tool [22], and the cross-sectional study was evaluated using the Joanna Briggs Institute (JBI) Critical Appraisal Checklist for Analytical Cross-Sectional Studies [23]. Disagreements were resolved through discussion and consensus or, when necessary, consultation with a third reviewer (HFGB).

### 2.7. Data Synthesis

Given the clinical and methodological heterogeneity across the included studies, a meta-analysis was not performed, and findings were synthesized narratively. Studies were grouped according to four prespecified neuropsychiatric outcome domains: depressive symptoms, anxiety symptoms, manic or hypomanic symptoms, and suicidal ideation or behavior. The narrative synthesis was structured with reference to the Synthesis Without Meta-analysis (SWiM) reporting guideline [24].

At the outcome level, depressive symptoms were assessed using symptom scales, adverse-event reporting, and coded clinical diagnoses, while anxiety was similarly captured using both standardized scales and psychiatric adverse events or diagnostic codes. Suicide-related outcomes varied from prospectively assessed C-SSRS events to BDI-II item scores and EHR-based composite diagnoses of suicidal ideation, suicide attempts, or self-harm, with markedly different comparators and follow-up periods. Manic or hypomanic symptoms were not systematically assessed and therefore provided no comparable estimates for quantitative synthesis (Table 2). These differences were particularly relevant for depressive and suicidal outcomes, which were assessed using non-equivalent clinical definitions and measurement approaches. Consequently, the available estimates did not represent sufficiently comparable clinical contrasts to support a single pooled effect estimate. Results were therefore synthesized by outcome domain, with emphasis on the direction and consistency of findings, study design, risk of bias, and potential sources of heterogeneity rather than statistical significance alone.

### 2.8. Certainty of Evidence Assessment

The certainty of evidence was assessed using the Grading of Recommendations Assessment, Development and Evaluation (GRADE) framework [25] for four prespecified neuropsychiatric outcome domains: depressive symptoms, anxiety symptoms, manic or hypomanic symptoms, and suicidal ideation or behavior. Certainty was assessed separately for each outcome domain rather than as a single overall rating for the review. For each outcome domain, the body of evidence was evaluated across the standard GRADE domains of risk of bias, inconsistency, indirectness, imprecision, and publication bias. Risk-of-bias judgments were informed by the design-specific assessments using RoB 2 for randomized trials, ROBINS-I for non-randomized longitudinal studies, and the JBI critical appraisal tool for the cross-sectional study. Inconsistency was assessed by considering the direction and magnitude of findings and the clinical and methodological heterogeneity across studies. Indirectness was evaluated in relation to the review question, including differences in study populations, GLP-1-based agents, comparators, outcome definitions and measurement methods, and follow-up duration. Imprecision was judged according to the amount of available evidence and the uncertainty surrounding the reported estimates.

Evidence from randomized and non-randomized studies was considered according to study design and methodological limitations when determining certainty for each outcome domain. Publication bias could not be formally assessed because the small number of studies contributing to individual outcome domains, together with heterogeneity in study designs, outcome definitions, and effect measures, precluded reliable funnel-plot-based assessment. The possibility of publication bias and selective outcome reporting therefore cannot be excluded.

## 3. Results

### 3.1. Study Selection

The study selection process is summarized in the PRISMA 2020 flow diagram (Figure 1). A total of 1964 records were identified through database searches, including 661 records from PubMed/MEDLINE, 865 from Scopus, and 438 from Web of Science. After removal of 580 duplicate records, 1384 records remained for title and abstract screening.

Following title and abstract screening, 1362 records were excluded, leaving 22 full-text articles for eligibility assessment. Eight articles were subsequently excluded because they did not meet the predefined eligibility criteria: seven did not meet the population criterion and one did not meet the outcome criterion. Ultimately, 14 studies were included in the qualitative synthesis.

### 3.2. Study Characteristics

A total of 14 studies were included, comprising four randomized studies (28.6%), nine longitudinal observational studies (64.3%), and one analytical cross-sectional study (7.1%). (Table 3). Detailed characteristics of the included studies according to study design are provided in Supplementary Tables S1–S3.

#### 3.2.1. Information on Randomized Clinical Trials

The studies were published between 2017 and 2026. The four randomized studies included adults with overweight or obesity, with or without type 2 diabetes depending on the study population. Both sexes were included, with women accounting for between 63% and 77.6% of participants. Depressive symptoms were assessed using the Patient Health Questionnaire-9 (PHQ-9) in all four studies (100%). Anxiety was not assessed using a dedicated standardized anxiety scale, and manic or hypomanic symptoms were not specifically assessed. Suicidal ideation and behavior were assessed using the Columbia-Suicide Severity Rating Scale (C-SSRS) in three studies (75.0%).

The GLP-1-based agents evaluated included liraglutide, semaglutide, and tirzepatide. Three studies (75.0%) used placebo comparators, whereas one study (25.0%) used an active comparator, directly comparing tirzepatide with semaglutide. Follow-up durations ranged from 32 to 160 weeks.

#### 3.2.2. Information from Observational Studies

The nine longitudinal observational studies were published between 2024 and 2026. All studies reported participant age, with mean or median ages ranging from 39.0 to 59.0 years. Study populations comprised adults with overweight or obesity, with type 2 diabetes present in some cohorts. Both sexes were represented; seven studies (77.8%) had predominantly female populations, whereas two (22.2%) had predominantly male populations. One study specifically enrolled military veterans.

Depressive outcomes were evaluated in five studies (55.6%) using either validated symptom scales, including the Hospital Anxiety and Depression Scale (HADS) and Beck Depression Inventory-II (BDI-II), or clinical outcomes derived from electronic health records. Anxiety outcomes were evaluated in two studies (22.2%), using HADS in one study and electronic health record-based clinical outcomes in the other. Manic or hypomanic symptoms were not specifically assessed in any of the longitudinal observational studies. Suicidal outcomes were evaluated in seven studies (77.8%) and were ascertained using the suicidal ideation item of the BDI-II or electronic health record/claims-based definitions of suicidal ideation, suicide attempts, self-harm, or composite suicidality outcomes.

The most frequently evaluated GLP-1-based agents were semaglutide (*n* = 7; 77.8%), liraglutide (*n* = 5; 55.6%), and tirzepatide (*n* = 4; 44.4%). Dulaglutide, exenatide, and lixisenatide were each evaluated in one study (*n* = 1; 11.1%). Follow-up durations and analytic time horizons varied substantially across studies, ranging from 4 months to 5 years.

#### 3.2.3. Information on the Cross-Sectional Study

The analytical cross-sectional study was published in 2024 and included 1105 adult women, with a mean age of 38.89 ± 9.00 years. The study population included women across different BMI categories, of whom 23.0% had overweight and 68.7% had obesity. Depressive symptoms were assessed using the Patient Health Questionnaire-9 (PHQ-9), whereas anxiety symptoms were evaluated using the Generalized Anxiety Disorder-7 (GAD-7). Manic or hypomanic symptoms and suicidal outcomes were not assessed. The GLP-1 receptor agonists evaluated were semaglutide and liraglutide.

### 3.3. Neuropsychiatric Outcomes

#### 3.3.1. Depressive Symptoms

Depressive outcomes were evaluated in ten of the fourteen included studies, comprising four randomized studies, five longitudinal observational studies, and one analytical cross-sectional study. In the randomized evidence, depressive symptoms were assessed using the Patient Health Questionnaire-9 (PHQ-9). In the liraglutide trial, mean PHQ-9 scores decreased from 2.8 ± 3.0 to 1.8 ± 2.7 in the liraglutide group and from 2.9 ± 3.1 to 1.9 ± 2.7 in the placebo group, with no meaningful between-group difference (estimated treatment difference: −0.02; 95% CI: −0.17 to 0.12) [26]. In the semaglutide trials, baseline PHQ-9 scores were 2.0 ± 2.3 and 1.8 ± 2.3 in the semaglutide and placebo groups, respectively, and were 2.0 ± 2.9 and 2.4 ± 3.3 at week 68. The estimated treatment difference was −0.56 points (95% CI: −0.81 to −0.32; *p* < 0.001), and semaglutide was associated with lower odds of progression to a more severe PHQ-9 category (OR: 0.63; 95% CI: 0.50–0.79; *p* < 0.001); however, the magnitude of the between-group difference in PHQ-9 scores was not considered clinically meaningful [14].

Similarly, in the placebo-controlled tirzepatide trial, mean PHQ-9 scores were 2.7 ± 3.0 versus 2.6 ± 3.1 at baseline and 1.9 ± 2.7 versus 2.4 ± 3.3 at week 72 for tirzepatide and placebo, respectively, with an estimated treatment difference of −0.6 points (SE 0.1; *p* < 0.001). Progression to a more severe PHQ-9 category occurred in 18.2% of tirzepatide treated participants compared with 24.3% receiving placebo (*p* < 0.001) [27]. In the head-to-head randomized comparison of tirzepatide and semaglutide, categorical PHQ-9 changes through week 72 were broadly similar between treatment groups, with most participants who had no or minimal depressive symptoms at baseline remaining within that category and transitions to higher-severity categories being uncommon [28].

Findings from longitudinal observational studies were more heterogeneous. After four months of liraglutide treatment, Kuckuck et al. reported a reduction in depressive symptoms measured using the HADS depression subscale (β = −0.97; 95% CI: −1.85 to −0.10; *p* = 0.0317) [17]. In a six-month pre–post study of veterans receiving semaglutide or tirzepatide, mean BDI-II scores decreased from 17.2 ± 10.4 to 12.0 ± 9.9 (*p* < 0.001), corresponding to a moderate effect size (Cohen’s d = 0.64; 95% CI: 0.29–0.97) [29].

In contrast to these symptom-scale studies, large electronic health record-based analyses evaluated incident clinical outcomes. In a target trial emulation among adults with obesity without diabetes, GLP-1 RA use was associated with a lower risk of depression compared with other anti-obesity medications (HR: 0.63; 95% CI: 0.61–0.65; *p* < 0.001) [30]. Notably, neuropsychiatric outcomes were secondary outcomes in this study, which was primarily designed to evaluate cardiovascular and kidney endpoints. Kornelius et al. reported a higher incidence of major depressive disorder among GLP-1 RA users compared with matched non-users, with cumulative incidences of 7.02% versus 2.68% at five years and an HR of 2.95 (95% CI: 2.82–3.08) [15]. Chang et al. similarly found a modestly higher incidence of a composite depression outcome among GLP-1 RA initiators compared with SGLT2 inhibitor initiators (17.0% vs. 14.8%; HR: 1.09; 95% CI: 1.04–1.14; *p* = 0.0001), corresponding to an absolute risk difference of 2.2% (95% CI: 1.5–2.8%) [33].

Finally, in the analytical cross-sectional study, women receiving semaglutide had lower mean PHQ-9 scores than women not receiving semaglutide (9.76 ± 6.37 vs. 10.84 ± 6.33; *p* = 0.013) [35]. Although statistically significant, the approximately one-point between-group difference was small, while mean scores in both groups reflected a clinically relevant depressive symptom burden. Therefore, the statistical difference should be interpreted cautiously in terms of clinical meaningfulness. Given the cross-sectional assessment of exposure and depressive symptoms, temporal direction cannot be established, and reverse causation cannot be excluded.

Overall, randomized studies consistently showed no clinically meaningful worsening of depressive symptoms with GLP-1-based therapy. Small statistically significant improvements in PHQ-9 scores were observed in some placebo-controlled trials, but these differences were below accepted thresholds for clinical relevance. In contrast, observational evidence remained inconsistent, with studies reporting improvements in depressive symptoms, lower incidence of depression, or conversely increased risks of incident depressive disorders in large electronic health record-based cohorts.

#### 3.3.2. Anxiety Symptoms

Anxiety-related outcomes were reported in six of the fourteen included studies, comprising three randomized studies, two longitudinal observational studies, and one analytical cross-sectional study. In the randomized evidence, unlike depressive symptoms, anxiety was not assessed using a dedicated standardized rating scale but was captured as a psychiatric adverse event during follow-up. O’Neil et al. reported similarly low rates of anxiety adverse events with liraglutide and placebo (1.9 vs. 1.7 events per 100 person-years) [26]. In the semaglutide trials, anxiety-related adverse events were broadly similar between treatment groups, occurring in 3.3% versus 4.2% of participants in STEP 1–3 and 7.2% versus 9.2% in STEP 5 for semaglutide and placebo, respectively [14]. In the placebo-controlled tirzepatide trials, anxiety symptoms were reported in 1.50% of tirzepatide-treated participants and 3.52% of those receiving placebo, whereas anxiety disorders not elsewhere classified were uncommon in both groups (0.18% vs. 0.08%) [27].

Among longitudinal observational studies, Kuckuck et al. assessed anxiety using the Hospital Anxiety and Depression Scale anxiety subscale (HADS-A) and observed a non-significant reduction after four months of liraglutide treatment (β = −0.67; 95% CI: −1.46 to 0.11; *p* = 0.095). Psychological well-being, assessed using the OBESI-Q Psychological Well-being scale, improved during the same period (β = +4.31; 95% CI: 0.81–7.81; *p* < 0.05) [17]. In contrast, Kornelius et al., using electronic health record-based diagnoses, reported a higher risk of anxiety among GLP-1 RA users compared with matched non-users (HR: 2.08; 95% CI: 2.04–2.12) [15].

The analytical cross-sectional study by Witaszek et al. evaluated anxiety symptoms using the Generalized Anxiety Disorder-7 (GAD-7). Mean GAD-7 scores were lower among semaglutide users than among non-users (8.71 ± 6.16 vs. 9.80 ± 6.07; *p* = 0.013) [35]. However, both mean scores were within the moderate anxiety range, indicating that although the between-group difference was statistically significant, its absolute magnitude was small in the context of the overall symptom burden. Given the cross-sectional design, temporal direction cannot be established, and reverse causation cannot be excluded.

Overall, randomized studies did not indicate an increased frequency of anxiety-related psychiatric adverse events with GLP-1-based therapy. Observational findings were less consistent, with a non-significant improvement in anxiety symptoms in a small prospective study contrasting with an increased risk of clinically recorded anxiety in a large retrospective cohort. The cross-sectional evidence suggested slightly lower anxiety symptom scores among semaglutide users, although the magnitude of the difference was small.

#### 3.3.3. Hypomanic/Manic Symptoms

Evidence regarding manic or hypomanic symptoms was extremely limited. None of the fourteen included studies prospectively assessed manic or hypomanic symptoms using standardized rating scales or as a prespecified clinical outcome. Although isolated bipolar/manic-spectrum events were captured within general psychiatric adverse-event reporting in randomized trials, their extremely low frequency precluded meaningful estimation or comparison of event rates. Therefore, the available evidence was insufficient to determine whether GLP-1-based therapies are associated with manic or hypomanic symptoms.

#### 3.3.4. Suicidal Ideation, Suicide Attempts and Self-Harm

Suicide-related outcomes were evaluated in ten of the fourteen included studies, comprising three randomized studies and seven longitudinal observational studies. In the randomized evidence, suicidal ideation and behavior were prospectively assessed using the Columbia-Suicide Severity Rating Scale (C-SSRS). In the liraglutide trials, suicidal ideation during treatment was reported in 34/3291 participants (1.0%) receiving liraglutide and 19/1843 (1.0%) receiving placebo. Adverse-event reporting identified a small numerical imbalance in suicidal ideation or behavior (0.3% vs. 0.1%), although prospective C-SSRS assessments did not show a between-group imbalance [26]. In the semaglutide trials, incident suicidal ideation occurred in 0.4% versus 0.6% of participants in STEP 1–3 and 0.7% versus 1.4% in STEP 5 for semaglutide and placebo, respectively. One suicide attempt was reported in a semaglutide-treated participant; because of the small number of events, no formal comparative hypothesis testing was performed [14]. In the tirzepatide trials, suicidal ideation occurred in 0.6% of participants in both treatment groups, while suicidal behavior was reported in 0.1% of tirzepatide-treated participants and none receiving placebo [27].

Among longitudinal observational studies, Rutledge et al. assessed suicidal ideation using item 9 of the Beck Depression Inventory-II (BDI-II). Among the 40 participants who completed six-month follow-up, mean scores were unchanged from baseline to post-treatment (0.08 ± 0.27 vs. 0.08 ± 0.27; *p* = 1.0; Cohen’s d = 0.00, 95% CI: −0.31 to 0.31) [29]. In a large target-trial emulation among adults with obesity without diabetes, GLP-1 RA use was associated with a lower risk of the composite outcome of suicidal ideation or attempt compared with other anti-obesity medications (HR: 0.42; 95% CI: 0.35–0.51; *p* < 0.001) [30]. In contrast, Kornelius et al. reported a higher cumulative incidence of suicidal ideation or attempts among GLP-1 RA users compared with matched non-users (3.64% vs. 1.42% at five years; HR: 2.06; 95% CI: 1.92–2.21) [15].

Hurtado et al. found no statistically significant increase in suicidal ideation or self-injury with GLP-1 RA use compared with SGLT2 inhibitors in the main per-protocol analysis (HR: 1.04; 95% CI: 0.35–3.14), with similarly imprecise results in intention-to-treat and sensitivity analyses. The wide confidence intervals reflected the rarity of events and remained compatible with both no effect and a clinically important increase in risk [31]. Her et al. likewise reported no statistically significant difference in incident suicidal ideation (RR: 1.36; 95% CI: 0.62–6.14) or suicidality (RR: 1.18; 95% CI: 0.57–3.63) among semaglutide users compared with active weight-management comparators; however, estimates were highly imprecise, and the authors noted potential under ascertainment of suicide-related outcomes using ICD-10 codes [34].

Yu et al. observed a lower risk of suicidal ideation or suicide attempts among tirzepatide users compared with non-GLP-1 anti-obesity medications (17 vs. 33 events after matching; aHR: 0.52; 95% CI: 0.28–0.91), although the observational design precludes causal inference [32]. Wang et al. similarly reported lower risks of both incident suicidal ideation (0.11% vs. 0.43%; HR: 0.27; 95% CI: 0.20–0.36) and recurrent suicidal ideation (HR: 0.44; 95% CI: 0.32–0.60) among semaglutide users compared with non-GLP-1 anti-obesity medications [16]. These large electronic health record-based estimates should nevertheless be interpreted cautiously because of methodological limitations inherent to observational analyses of rare psychiatric outcomes, including potential residual confounding, confounding by indication, time-zero and censoring choices, outcome ascertainment, and reverse causation.

Overall, randomized studies showed very low frequencies of suicidal ideation or behavior and no consistent imbalance between GLP-1-based therapy and placebo. Observational findings were heterogeneous, ranging from lower observed risks in several large database studies to no clear association in imprecise cohort analyses and a higher risk in one large retrospective cohort. Given the rarity of events and the methodological limitations of observational data, the available evidence does not support a definitive causal association in either direction.

### 3.4. Risk of Bias Assessment

The risk-of-bias assessment is summarized in Supplementary Figures S1 and S2 and Supplementary Table S4. Application of the RoB 2 tool to the four randomized studies resulted in an overall judgment of some concerns for all studies. The randomization process, deviations from intended interventions, and missing outcome data were consistently judged to be at low risk of bias. Some concerns arose primarily from selection of the reported result in three studies and from outcome measurement in one study (Supplementary Figure S1).

Assessment of the nine longitudinal observational studies using the ROBINS-I tool showed heterogeneous methodological quality. Four studies were judged to have a serious overall risk of bias and five a moderate overall risk. Confounding was the principal source of serious risk, with additional concerns related to participant selection, deviations from intended interventions, and missing data. Classification of interventions and selection of the reported result were consistently judged to be at low risk of bias (Supplementary Figure S2).

The single analytical cross-sectional study assessed using the Joanna Briggs Institute (JBI) Critical Appraisal Checklist demonstrated an overall low risk of bias. The study adequately defined the inclusion criteria, clearly described the study population, used valid and reliable exposure and outcome measurements, identified potential confounding factors, and employed appropriate statistical analyses (Supplementary Table S4).

### 3.5. GRADE Certainty of Evidence

GRADE was applied at the outcome-domain level rather than as a single overall certainty rating for the review. Randomized evidence was initially rated as high certainty, whereas observational evidence was initially rated as low certainty, with subsequent assessment across the GRADE domains of risk of bias, inconsistency, indirectness, imprecision, and publication bias. Given the narrative synthesis and the inclusion of different study designs, certainty judgments considered the overall body of evidence contributing to each prespecified outcome. The complete GRADE evidence profile is presented in Table 4. Certainty of evidence was LOW for depressive symptoms and VERY LOW for anxiety symptoms and suicide-related outcomes, primarily because of concerns regarding risk of bias, inconsistency across studies, and, for anxiety and suicide-related outcomes, imprecision. GRADE was not applied to manic/hypomanic symptoms because no included study systematically assessed this outcome.

## 4. Discussion

This systematic review synthesized the available evidence on neuropsychiatric outcomes associated with GLP-1-based therapies in adults with overweight or obesity across 14 studies, including four randomized studies, nine longitudinal observational studies, and one analytical cross-sectional study. Overall, the available evidence did not demonstrate a consistent pattern of increased neuropsychiatric risk. Randomized studies generally showed no clinically meaningful worsening of depressive symptoms and low frequencies of suicide-related events, whereas observational findings were more heterogeneous, with associations ranging from symptom improvement or lower observed risks to neutral findings and increased risks for some outcomes. Importantly, these findings should be interpreted considering the certainty of the available evidence, which was rated as low for depressive symptoms and very low for anxiety and suicide-related outcomes; manic or hypomanic symptoms could not be graded because they were not systematically assessed in the included studies. Accordingly, the current evidence does not permit definitive conclusions regarding either neuropsychiatric harm or benefit, particularly given the methodological limitations and heterogeneity of the observational evidence.

Regarding depressive outcomes, randomized evidence consistently showed no clinically meaningful worsening of depressive symptoms with GLP-1-based therapies. Although statistically significant differences favoring semaglutide and tirzepatide were observed in some trials, the magnitude of change in PHQ-9 scores was small, and categorical changes generally favored maintenance of no or minimal symptoms rather than indicating a clinically established antidepressant effect [14,26,27,28]. Improvements were also observed in longitudinal studies using HADS-D and BDI-II [17,29], raising the possibility that GLP-1-based treatment may be accompanied by improvement in affective symptoms. However, these findings cannot establish a direct antidepressant effect, as changes in body weight, metabolic health, physical functioning, or other aspects of treatment may contribute to the observed improvements. In contrast, large observational studies produced conflicting estimates: lower incident depression was reported in one target-trial emulation [30], whereas higher risks were observed in other electronic health record-based cohorts [15,33]. Such discrepancies may partly reflect residual confounding and confounding by indication, as well as surveillance and detection bias, particularly when incident depression is identified through diagnostic codes rather than standardized prospective psychiatric assessments. These biases may increase the likelihood of detecting previously unrecognized psychiatric conditions among patients receiving more intensive clinical follow-up, although they cannot be assumed to fully explain the increased-risk estimates. The cross-sectional findings of Witaszek et al. should likewise be interpreted cautiously: despite statistically lower PHQ-9 scores among semaglutide users, the between-group difference was small within a population with clinically relevant depressive symptom burden, and the cross-sectional design precludes establishing temporality and remains susceptible to reverse causation [35]. Overall, the randomized evidence is reassuring regarding clinically meaningful worsening of depressive symptoms, but evidence for a specific antidepressant benefit remains insufficient, while conflicting observational findings limit causal inference.

For anxiety-related outcomes, the randomized evidence did not suggest an increased frequency of anxiety with GLP-1-based therapies; however, unlike depressive symptoms, anxiety was captured as a psychiatric adverse event rather than prospectively assessed using dedicated standardized symptom scales [14,26,27]. This distinction limits conclusions regarding potential changes in anxiety symptom severity. Among observational studies, Kuckuck et al. found a non-significant reduction in HADS-A scores after liraglutide treatment, despite improvement in broader psychological well-being [17], whereas Kornelius et al. reported a substantially higher risk of clinically recorded anxiety among GLP-1 RA users in a large retrospective cohort [15]. These divergent findings may partly reflect differences in outcome ascertainment, study design, comparator selection, and residual confounding, particularly when diagnostic codes are used to identify incident anxiety. Witaszek et al. reported statistically lower GAD-7 scores among semaglutide users; however, the absolute between-group difference was small, both groups had clinically relevant anxiety symptom burden, and the cross-sectional design precludes establishing temporality or excluding reverse causation [35]. Taken together, the available evidence does not indicate a consistent anxiety-related safety signal, but it also provides insufficient support for an anxiolytic effect, particularly given the very low certainty of evidence and the limited prospective assessment of anxiety symptoms.

Evidence regarding manic or hypomanic symptoms was particularly limited. None of the included studies systematically assessed these symptoms using dedicated standardized rating scales. Available information was restricted to isolated bipolar/manic-spectrum events captured within general psychiatric adverse-event reporting in randomized trials. Such findings cannot establish the incidence of treatment-emergent mania or hypomania or support meaningful comparisons between GLP-1-based therapies and control groups. Accordingly, GRADE was not applied to this outcome, and the current evidence is insufficient to determine whether GLP-1-based therapies influence the risk of manic or hypomanic symptoms.

Suicide-related outcomes were uncommon in the randomized studies, with no consistent imbalance in suicidal ideation or behavior between GLP-1-based therapies and placebo when prospectively assessed using the C-SSRS [14,26,27]. Although these findings are reassuring, the low number of events limits the precision of comparative estimates and does not allow small but clinically relevant differences in risk to be excluded. Observational evidence was substantially more heterogeneous. Suicidal ideation remained unchanged in a small pre–post study using the BDI-II [29], while lower observed risks of suicidal ideation or attempts were reported in several large database studies [16,30,32]. In contrast, Hurtado et al. and Her et al. found no statistically significant associations, but their estimates were highly imprecise and remained compatible with clinically important differences in risk [31,34], whereas Kornelius et al. reported a higher risk of suicidal ideation or attempts among GLP-1 RA users [15]. These divergent estimates should be interpreted cautiously because observational analyses of rare psychiatric outcomes remain vulnerable to residual confounding, confounding by indication, differences in outcome ascertainment, time-zero and censoring choices, and reverse causation. This is particularly relevant to the large electronic health record analysis by Wang et al., in which the magnitude of the observed associations should not be interpreted as evidence of a causal protective effect [16]. Overall, the available evidence does not demonstrate a consistent suicide-related safety signal in either direction, and the very low certainty of evidence precludes definitive causal conclusions.

Our findings are broadly consistent with the recent systematic review and meta-analysis by Pierret et al. [36], which included randomized double-blind placebo-controlled trials in adults with overweight/obesity and/or diabetes and found no increased risk of serious or non-serious psychiatric adverse events and no significant worsening of depressive symptoms with GLP-1 RA treatment. Pierret et al. additionally reported modest improvements in mental health-related quality of life and eating-related outcomes, supporting the possibility that the psychiatric effects of GLP-1-based therapies may extend beyond the absence of harm. However, evidence for anxiety and suicidality in that review was insufficient for quantitative synthesis. The present review complements these findings by focusing specifically on adults with overweight or obesity and integrating recent randomized and observational evidence across depressive symptoms, anxiety, manic/hypomanic symptoms, and suicide-related outcomes. This broader evidence base identifies potential improvements in affective symptoms in some studies but also reveals substantial heterogeneity across observational estimates. Importantly, these apparent benefits should not be interpreted as evidence of an antidepressant or anxiolytic effect, as the observational evidence was generally of lower certainty and vulnerable to residual confounding, selection bias, and reverse causation.

Potential heterogeneity across GLP-1–based therapies should also be considered when interpreting the present findings. The included studies evaluated different agents, predominantly liraglutide, semaglutide, and tirzepatide, which differ in molecular structure, pharmacokinetic properties, and receptor activity. Available evidence also suggests that CNS access and engagement may vary among individual GLP-1 receptor agonists; however, direct evidence of CNS penetration in humans remains limited, and whether such differences are clinically meaningful has not been established [37]. In addition, tirzepatide is a dual GIP/GLP-1 receptor agonist and therefore should not necessarily be assumed to share identical neuropsychiatric effects with selective GLP-1 receptor agonists. Nevertheless, the available clinical evidence remains insufficient to establish agent-specific differences in psychiatric outcomes, as individual drugs were evaluated across different study designs, populations, comparators, and follow-up periods. Accordingly, the present findings should not be interpreted as demonstrating a uniform class effect or as establishing differential psychiatric safety or benefit among individual agents.

Beyond psychiatric safety, some of the included studies suggested potential improvements in depressive or anxiety symptoms during GLP-1–based treatment. Several mechanisms could plausibly contribute to these observations, including weight loss, improved metabolic health and physical functioning, as well as potential central effects of GLP-1 receptor signaling. However, the relative contribution of these pathways remains uncertain. Although preclinical and neuroimaging evidence supports CNS engagement for some GLP-1 receptor agonists, direct evidence of CNS penetration and target engagement in humans remains limited, and peripheral mechanisms may also contribute to observed CNS-related effects [37]. Accordingly, the improvements observed in some studies should not be interpreted as evidence of a direct antidepressant or anxiolytic effect, particularly given the predominance of observational evidence and the low or very low certainty of evidence for these outcomes.

### Limitations and Future Directions

Several limitations should be acknowledged. The available evidence was characterized by substantial clinical and methodological heterogeneity, as detailed in Section 2.7, limiting direct comparability across studies. Much of the evidence was observational, and several longitudinal studies were judged to have serious risk of bias, particularly because of confounding, participant selection, deviations from intended interventions, and missing data; consequently, these associations should not be interpreted causally. Psychiatric outcomes were also assessed inconsistently, ranging from validated symptom scales to psychiatric adverse-event reporting and ICD-coded diagnoses, introducing potential differences in sensitivity and susceptibility to surveillance bias, detection bias, and outcome misclassification. Suicide-related events were uncommon, resulting in substantial imprecision, while manic or hypomanic symptoms were not systematically assessed. In addition, randomized trials generally excluded individuals with known major psychopathology, limiting generalizability to patients with pre-existing or more severe psychiatric disorders.

Additional limitations relate to the scope of the evidence base. The search was restricted to PubMed/MEDLINE, Scopus, and Web of Science, to publications in English or Spanish, and to the predefined publication period; unpublished and grey literature were not systematically searched, and publication bias therefore cannot be excluded. Eating-disorder outcomes were outside the prespecified scope of this review and were not evaluated, representing an important evidence gap given their clinical relevance in populations receiving pharmacological treatment for obesity. Furthermore, although several GLP-1–based therapies were included, differences in pharmacology, populations, comparators, and available evidence prevented reliable molecule-specific comparisons, and treating these agents together at some levels of the synthesis may have obscured agent-specific signals. Future studies should therefore prioritize prospective assessment using standardized psychiatric instruments, adequately powered evaluation of rare outcomes such as suicidal behavior and manic or hypomanic symptoms, inclusion of individuals with pre-existing psychiatric disorders, and direct comparisons across individual GLP-1–based therapies. Future research should also specifically evaluate eating-disorder outcomes and clarify whether observed changes in affective symptoms represent direct treatment effects or are mediated by weight loss, metabolic improvement, or other factors.

## 5. Conclusions

Current evidence does not demonstrate a consistent increase in depressive symptoms, anxiety, or suicide-related outcomes associated with GLP-1–based therapies in adults with overweight or obesity. Randomized studies generally showed no consistent imbalance in psychiatric outcomes between active treatment and placebo; however, these outcomes were frequently secondary or safety endpoints, events such as suicidal behavior were uncommon, and participants with major pre-existing psychopathology were often excluded. Observational findings were more heterogeneous, with estimates ranging from potential benefit to no association and increased risk and should be interpreted considering their methodological limitations.

Some studies suggested improvements in depressive or anxiety symptoms, but the available evidence is insufficient to establish a direct antidepressant or anxiolytic effect. Certainty of evidence was low for depressive symptoms and very low for anxiety and suicide-related outcomes, while evidence for manic or hypomanic symptoms was insufficient for GRADE assessment. Differences among individual GLP-1–based therapies, study populations, baseline psychiatric comorbidity, and outcome ascertainment further limit class-wide conclusions. Prospective studies using standardized psychiatric assessments, including individuals with pre-existing psychiatric disorders and adequately powered for rare outcomes, are needed to clarify both potential benefits and risks and to determine whether neuropsychiatric effects differ among individual agents.

## Figures and Tables

**Table 1:** Eligibility criteria.

Inclusion Criteria	Exclusion Criteria
1. Adults aged ≥ 18 years with overweight or obesity (BMI ≥ 25 kg/m^2^), with or without T2DM.	1. Children or adolescents (<18 years) and populations not meeting the overweight or obesity criterion.
2. Exposure to a GLP-1 RA (semaglutide, liraglutide, dulaglutide, exenatide, lixisenatide, or albiglutide) or the dual GIP/GLP-1 receptor agonist tirzepatide.	2. Animal, preclinical, or in vitro studies; reviews, editorials, letters, conference abstracts, protocols, and case reports/series.
3. Studies evaluating ≥1 prespecified neuropsychiatric outcome: depressive symptoms, anxiety symptoms, manic/hypomanic symptoms, suicidal ideation, suicide attempts, or self-harm.	3. Studies without identifiable exposure to an eligible GLP-1-based treatment.
4. Randomized or non-randomized clinical trials and observational studies, including cohort, case–control, and cross-sectional designs.	4. Studies focused exclusively on metabolic, glycemic, cardiovascular, hepatic, or weight-loss outcomes without assessment of a prespecified neuropsychiatric outcome.
5. Full-text articles published in English or Spanish during the 10-year period covered by the search.	5. Studies primarily evaluating GLP-1-based therapies as treatment for pre-existing psychiatric disorders rather than assessing the prespecified neuropsychiatric outcomes in adults with overweight or obesity.
	6. Duplicate publications or studies with insufficient data for extraction.

**Table 2:** Main sources of heterogeneity across included studies.

Dimension	Variability Across Studies
**Study design**	Randomized studies (*n* = 4); longitudinal observational studies (*n* = 9); cross-sectional study (*n* = 1).
**GLP-1 RA exposure**	Single GLP-1 RA evaluated (*n* = 7); multiple GLP-1 RAs evaluated (*n* = 7).
**Comparator structure**	Placebo (*n* = 3); active pharmacological comparator (*n* = 7); non-exposed comparator (*n* = 1); within-person baseline (*n* = 2); cross-sectional exposure comparison (*n* = 1).
**Outcome ascertainment**	Standardized psychiatric/psychological instruments (*n* = 7); predominantly EHR/claims-based clinical definitions or coded outcomes (*n* = 7).
**Follow-up**	Cross-sectional assessment (*n* = 1); longitudinal follow-up ranging from 4 months to 5 years.

**Table 3:** Summary of the characteristics and main neuropsychiatric outcomes of the included studies.

**Randomized Controlled Trials (*n* = 4)**					
**Study**	**Clinical Population**	**Group Comparison (Dose, N)**	**Follow-Up**	**Psychiatric Outcome (Instrument)**	**Main Quantitative Findings**
**O’Neil et al. 2017 [26]**	Adults with overweight/obesity (±T2DM)	GLP-1 RA: Liraglutide 3.0 mg (*n* = 3384) Comparator: Placebo (*n* = 1941)	32–160 weeks	Depression (PHQ-9), suicidal ideation (C-SSRS), psychiatric AEs	No between-group differences in PHQ-9 (MD −0.02; 95% CI −0.17 to 0.12) or C-SSRS suicidal ideation (1.0% vs. 1.0%). Similar depression and anxiety AE rates; numerical imbalance in suicide-related AEs (0.3% vs. 0.1%).
**Wadden et al., 2024 [14]**	Adults with overweight/obesity (±T2DM) without major psychopathology	GLP-1 RA: Semaglutide 2.4 mg (*n* = 2268) Comparator: Placebo (*n* = 1413)	68–104 weeks	Depression (PHQ-9), suicidal ideation/behavior (C-SSRS), psychiatric AEs	PHQ-9 scores remained low in both groups (MD −0.56; 95% CI −0.81 to −0.32). No increased risk of suicidal ideation/behavior or psychiatric AEs versus placebo.
**Wadden et al., 2026 [27]**	Adults with overweight/obesity (±T2DM) without major psychopathology	GLP-1 RA: Tirzepatide 5/10/15 mg (*n* = 2806) Comparator: Placebo (*n* = 1250)	72 weeks	Depression (PHQ-9), suicidal ideation/behavior (C-SSRS), psychiatric AEs	PHQ-9 scores remained low in both groups (LSM difference −0.6; *p* < 0.001). No increased risk of suicidal ideation (0.6% vs. 0.6%) or psychiatric AEs versus placebo.
**Shukla et al., 2026 [28]**	Adults with overweight/obesity without T2DM	GLP-1 RA: Tirzepatide MTD (10/15 mg) (*n* = 374) Comparator: Semaglutide MTD (1.7/2.4 mg) (*n* = 376)	72 weeks	Depressive symptoms (PHQ-9); mental HRQoL (SF-36 MCS)	PHQ-9 category shifts were similar between groups, with no increase in depressive symptoms. Mental health (SF-36 MCS) did not differ between tirzepatide and semaglutide.
**Observational Studies (*n* = 9)**					
**Study**	**Clinical Population**	**Group Comparison (Dose, N)**	**Follow-Up**	**Psychiatric Outcome (Instrument)**	**Main Quantitative Findings**
**Kuckuck et al., 2026 [17]**	Adults with obesity	GLP-1 RA: Liraglutide 3.0 mg (*n* = 98) Comparator: Baseline	4 months	Depression and anxiety (HADS); psychological wellbeing (ObesiQ)	Liraglutide was associated with reduced HADS total (β −1.65; 95% CI −3.19 to −0.11) and depression scores (β −0.97; 95% CI −1.85 to −0.10), with improved psychological wellbeing (β 4.31; 95% CI 0.81–7.81). No deterioration in mental health was observed.
**Rutledge et al., 2026 [29]**	Veterans with obesity and metabolic comorbidities	GLP-1 RA: Semaglutide or tirzepatide (dose NR; *n* = 40) Comparator: Baseline	6 months	Depression (BDI-II), suicidal ideation, PTSD, psychological distress	Significant reductions in depressive symptoms (BDI-II: −30%; *p* < 0.001) and improvements in general mental health, with no increase in suicidal ideation.
**Tang et al., 2025 [30]**	Adults with obesity without T2DM	GLP-1 RA: (liraglutide, semaglutide, or tirzepatide; dose NR; *n* = 140,169). Comparator: Other anti-obesity medications (*n* = 140,169)	Mean follow-up: 392 days for GLP-1 RA users	Depression; suicidal ideation/attempt (EHR/ICD-10-based outcomes)	GLP-1 RA use was associated with a lower risk of depression (HR 0.63; 95% CI 0.61–0.65) and suicidal ideation/attempt (HR 0.42; 95% CI 0.35–0.51).
**Kornelius et al., 2024 [15]**	Adults with obesity (BMI ≥ 30 kg/m^2^)	GLP-1 RA: (liraglutide 1.8/3.0 mg; semaglutide 1.0/2.4 mg; *n* = 162,253) Comparator: Patients with obesity not receiving GLP-1 RAs (*n* = 162,253)	6 months–5 years	Depression, anxiety, suicidal ideation/attempts (ICD-10)	GLP-1 RA use was associated with a higher risk of any psychiatric disorder (HR 1.98; 95% CI 1.94–2.01), major depression (HR 2.95; 95% CI 2.82–3.08), anxiety (HR 2.08; 95% CI 2.04–2.12), and suicidal ideation/attempts (HR 2.06; 95% CI 1.92–2.21).
**Hurtado et al., 2024 [31]**	Adults with obesity (BMI ≥ 30 kg/m^2^ or obesity diagnosis) and T2DM	GLP-1 RA: (dose NR; *n* = 3040) Comparator: SGLT2 inhibitors (*n* = 11,627)	Mean follow-up: 483 days	Suicidal ideation and self-injury (ICD-9/10)	No increased risk of suicidal ideation or self-injury compared with SGLT2 inhibitors (HR 1.04; 95% CI 0.35–3.14).
**Wang et al., 2024 [16]**	Adults with overweight or obesity	GLP-1 RA: Semaglutide (dose NR; *n* = 52,783) Comparator: Non-GLP-1 anti-obesity medications (bupropion, naltrexone, orlistat, topiramate, phentermine, setmelanotide; *n* = 52,783)	6 months	Incident and recurrent suicidal ideation (ICD codes/EHR)	Semaglutide was associated with a lower risk of incident suicidal ideation (HR 0.27; 95% CI 0.20–0.36) and recurrent suicidal ideation (HR 0.44; 95% CI 0.32–0.60) compared with non-GLP-1 anti-obesity medications.
**Yu et al., 2025 [32]**	Adults with overweight or obesity	GLP-1 RA: Tirzepatide (dose NR; *n* = 16,321) Comparator: Non-GLP-1 anti-obesity medications (*n* = 16,321)	Median: 365 days	Suicidal ideation or suicide attempts (EHR/ICD)	Tirzepatide was associated with a lower risk of suicidal ideation or suicide attempts (aHR 0.52; 95% CI 0.28–0.91)
**Chang et al., 2026 [33]**	Adults with overweight or obesity and newly diagnosed T2DM, without prior mood disorders	GLP-1 RA: tirzepatide, semaglutide, or liraglutide (dose NR; *n* = 25,704) Comparator: SGLT2 inhibitors (*n* = 25,704)	1 year	Incident depression: new diagnosis or antidepressant initiation (ICD-10/ATC)	GLP-1 RAs were associated with a higher incidence of depression than SGLT2 inhibitors (17.0% vs. 14.8%; HR 1.09, 95% CI 1.04–1.14).
**Her et al., 2025 [34]**	Adults with overweight or obesity without T2DM	GLP-1 RA: Semaglutide (dose NR; *n* = 16,822) Comparator: Active weight-management medications (phentermine, phentermine/topiramate, bupropion/naltrexone, orlistat; *n* = 11,986)	183 days	Incident suicidal ideation and suicidality (ICD-10)	No increased risk of suicidal ideation (RR 1.36, 95% CI 0.62–6.14) or suicidality (RR 1.18, 95% CI 0.57–3.63).
**Cross-Sectional Study (*n* = 1)**					
**Study**	**Clinical Population**	**Group Comparison (Dose, N)**		**Psychiatric Outcome (Instrument)**	**Main Quantitative Findings**
**Witaszek et al., 2024 [35]**	Adult women with overweight or obesity	GLP-1 RA: Semaglutide (*n* = 232), liraglutide (*n* = 235), naltrexone/bupropion (*n* = 41) Comparator: Participants not receiving the respective anti-obesity medication		Depression (PHQ-9) and anxiety (GAD-7)	Semaglutide use was associated with lower PHQ-9 (9.76 vs. 10.84, *p* = 0.013) and GAD-7 (8.71 vs. 9.80, *p* = 0.013) scores; no significant associations were observed with liraglutide or naltrexone/bupropion.

**Abbreviations:** AE, adverse event; BDI-II, Beck Depression Inventory-II; C-SSRS, Columbia-Suicide Severity Rating Scale; GAD, generalized anxiety disorder; GAD-7, Generalized Anxiety Disorder-7; GLP-1 RA, glucagon-like peptide-1 receptor agonist; HADS, Hospital Anxiety and Depression Scale; HRQoL, health-related quality of life; ICD, International Classification of Diseases; MCS, Mental Component Summary; NR, not reported; PHQ-9, Patient Health Questionnaire-9; PTSD, post-traumatic stress disorder; SF-36 MCS, 36-Item Short Form Health Survey Mental Component Summary;T2DM, type 2 diabetes mellitus.

**Table 4:** GRADE evidence profile—GLP-1-based therapies and neuropsychiatric outcomes.

Outcome	Study Design (# Studies)	Risk of Bias	Inconsistency	Indirectness	Imprecision	Publication Bias	Downgrading Domains	GRADE Certainty	Key Finding
**Depressive symptoms**	4 RCTs; 5 longitudinal observational; 1 cross-sectional (10 studies)	Serious (−1)	Serious (−1)	Not serious	Not serious	Could not be formally assessed	Risk of bias (−1); inconsistency (−1)	⊕⊕○○ LOW	RCTs showed no clinically meaningful worsening; observational findings were inconsistent.
**Anxiety symptoms**	3 RCTs; 2 longitudinal observational; 1 cross-sectional (6 studies)	Serious (−1)	Serious (−1)	Not serious	Serious (−1)	Could not be formally assessed	Risk of bias (−1); inconsistency (−1); imprecision (−1)	⊕○○○ VERY LOW	RCTs showed no increased anxiety-related psychiatric AEs, whereas observational evidence was inconsistent.
**Hypomanic/manic symptoms**	No studies specifically assessed the outcome	Not applicable	Not applicable	Not applicable	Not applicable	Not applicable	Not applicable	Not graded	Insufficient evidence; no study systematically assessed manic or hypomanic symptoms.
**Suicidal ideation, suicide attempts and self-harm**	3 RCTs; 7 longitudinal observational studies (10 studies)	Serious (−1)	Serious (−1)	Not serious	Serious (−1)	Could not be formally assessed	Risk of bias (−1); inconsistency (−1); imprecision (−1)	⊕○○○ VERY LOW	RCTs showed rare suicide-related events without a consistent treatment imbalance, while observational findings were heterogeneous and inconclusive.

⊕⊕○○ = LOW; ⊕○○○ = VERY LOW. NOTE GRADE was not applied to manic/hypomanic symptoms because no included study systematically assessed this outcome; isolated events reported within general psychiatric adverse-event surveillance were considered insufficient for meaningful certainty assessment.

## Data Availability

No new data were created or analyzed in this study. Data sharing is not applicable to this article.

## Supplementary Materials

The following supporting information can be downloaded at: https://www.mdpi.com/article/10.3390/brainsci16090926/s1: Supplementary Table S1. Characteristics of randomized controlled trials included in the systematic review; Supplementary Table S2. Characteristics of observational cohort studies included in the systematic review; Supplementary Table S3. Characteristics of the cross-sectional study included in the systematic review. Supplementary Table S4. Risk of bias assessment according to Methodological quality assessment of the cross-sectional study using the Joanna Briggs Institute (JBI) Critical Appraisal Checklist for Analytical Cross-Sectional Studies; Supplementary Table S5. Complete electronic search strategies used for the systematic review; Supplementary Table S6. PRISMA 2020 Main Checklist. Supplementary Figure S1. Risk of bias assessment according to the RoB 2 tool; Supplementary Figure S2. Risk of bias assessment according to the ROBINS-I tool.

## Abbreviations

The following abbreviations are used in this manuscript:

AE	Adverse Event
aHR	Adjusted Hazard Ratio
ATC	Anatomical Therapeutic Chemical
BDI-II	Beck Depression Inventory-II
BMI	Body Mass Index
C-SSRS	Columbia-Suicide Severity Rating Scale
CI	Confidence Interval
CNS	Central Nervous System
EHR	Electronic Health Record
EMA	European Medicines Agency
FDA	U.S. Food and Drug Administration
GAD-7	Generalized Anxiety Disorder-7
GIP	Glucose-Dependent Insulinotropic Polypeptide
GLP-1	Glucagon-Like Peptide-1
GLP-1 RA	Glucagon-Like Peptide-1 Receptor Agonist
GRADE	Grading of Recommendations Assessment, Development and Evaluation
HADS	Hospital Anxiety and Depression Scale
HADS-A	Hospital Anxiety and Depression Scale—Anxiety
HADS-D	Hospital Anxiety and Depression Scale—Depression
HR	Hazard Ratio
HRQoL	Health-Related Quality of Life
ICD	International Classification of Diseases
JBI	Joanna Briggs Institute
MCS	Mental Component Summary
MD	Mean Difference
MDD	Major Depressive Disorder
MeSH	Medical Subject Headings
NR	Not Reported
OR	Odds Ratio
PHQ-9	Patient Health Questionnaire-9
PRISMA	Preferred Reporting Items for Systematic Reviews and Meta-Analyses
PROSPERO	International Prospective Register of Systematic Reviews
PTSD	Post-Traumatic Stress Disorder
RCT	Randomized Controlled Trial
ROBINS-I	Risk Of Bias In Non-randomized Studies of Interventions
RoB 2	Risk of Bias 2
RR	Relative Risk
SE	Standard Error
SF-36 MCS	36-Item Short Form Health Survey Mental Component Summary
SGLT2	Sodium-Glucose Cotransporter 2
SGLT2i	Sodium-Glucose Cotransporter-2 Inhibitor
SWiM	Synthesis Without Meta-analysis
T2DM	Type 2 Diabetes Mellitus
