# Supplementary material for: Neuropsychiatric Outcomes Associated with GLP-1 Receptor Agonists in Adults with Overweight or Obesity: A Systematic Review
Source: Brain Sci. 2026 Aug 31;16(9):926. doi: 10.3390/brainsci16090926 (PMC13604221; doi:10.3390/brainsci16090926)
Supplement: Supplementary file 1 [file brainsci-16-00926-s001.zip › Supplementary Table S1.pdf]

Supplementary Table S1. Characteristics of randomized controlled trials included in the systematic review

First author, year	Country/setting	Design	Study objective	Population	Sociodemographic and clinical characteristics	Intervention	Comparator	Follow-up and assessment schedule	Neuropsychiatric outcomes evaluated
**O'Neil et al., 2017**	Multicenter international	Post hoc pooled analysis of five randomized, double-blind, placebo-controlled phase 2 and 3a trials	Evaluate neuropsychiatric safety of liraglutide 3.0 mg	Adults with BMI ≥30 kg/m², or BMI ≥27 kg/m² with ≥1 weight-related comorbidity.	N = 5,325; mean age 46.6 years; 71.8% female; mean BMI 37.9 kg/m² (liraglutide) and 37.8 kg/m² (placebo). History of psychiatric disorder: 13.4% vs 15.8%; depression: 9.1% vs 10.6%; anxiety: 7.2% vs 7.8%, respectively.	Once-daily subcutaneous liraglutide 3.0 mg (N = 3,384), titrated from 0.6 mg in weekly 0.6-mg increments; combined with reduced-calorie diet and exercise.	Placebo (N = 1,941), plus the same reduced-calorie diet and exercise intervention.	32–160 weeks; PHQ-9 and C-SSRS assessed throughout treatment; 12-week off-treatment follow-up in most trials.	Outcomes were assessed through adverse-event reporting (MedDRA), PHQ-9 for depressive symptoms, and C-SSRS for suicidal ideation and behavior.
**Wadden et al., 2024 (STEP)**	Multicenter international	Post hoc analysis of randomized placebo-controlled phase 3 trials	Evaluate psychiatric safety of semaglutide 2.4 mg in obesity	Adults with overweight/obesity ± DM2	N = 3,681: 3,377 in pooled STEP 1–3 and 304 in STEP 5. Mean age 49 and 47 years; female 69.9% and 77.6%, respectively. Mean BMI approximately 37–39 kg/m².	Subcutaneous semaglutide 2.4 mg once weekly (STEP 1–3: N = 2,116; STEP 5: N = 152), plus lifestyle intervention.	Placebo (STEP 1–3: N = 1,261; STEP 5: N = 152), plus lifestyle intervention.	68 weeks (STEP 1–3) and 104 weeks (STEP 5); PHQ-9 and C-SSRS assessed througho	Depression (PHQ-9), suicidal ideation/behavior (C-SSRS)

					Hypertension and dyslipidemia were common comorbidities.			ut treatment	
**Wadden et al., 2026 (SURMOUNT)**	Multicenter international	Post hoc analysis of randomized placebo-controlled phase 3 trials	To assess psychiatric safety of tirzepatide in adults with overweight or obesity without known major psychopathology.	Adults with BMI ≥30 kg/m² or ≥27 kg/m² with ≥1 weight-related comorbidity; SURMOUNT-2 included participants with T2D.	N = 4,056; mean age 47.2 years; 63.0% female; 74.0% White; mean BMI 37.3 kg/m²; mean obesity duration 15.3 years. Hypertension (40.4%) and dyslipidemia (36.5%) were common.	Once-weekly subcutaneous tirzepatide 5, 10, or 15 mg, or maximum tolerated dose 10/15 mg (N = 2,806), plus diet and physical activity modification.	Placebo (N = 1,250), plus diet and physical activity modification.	72 weeks; PHQ-9 assessed periodically and C-SSRS every 4 weeks, plus 4-week off-treatment safety follow-up.	Depression (PHQ-9), suicidal ideation/behavior (C-SSRS)
**Shukla et al., 2026**	Multicenter; United States	Phase 3b, multicenter, randomized, parallel-arm, open-label, active-controlled trial (SURMOUNT-5).	To evaluate changes in health-related quality of life, including depressive symptoms, with tirzepatide versus semaglutide.	Adults ≥18 years with BMI ≥30 kg/m², or BMI ≥27 kg/m² with ≥1 weight-related complication, without diabetes.	N = 750; mean age 44.7 years; 64.7% female; 76.1% White; mean BMI 39.4 kg/m²; mean body weight 113.0 kg; 56.7% had prediabetes; 50.1% had ≥2 obesity-related complications.	Once-weekly tirzepatide, maximum tolerated dose (10 or 15 mg), N = 374.	Once-weekly semaglutide, maximum tolerated dose (1.7 or 2.4 mg), N = 376.	72 weeks. PHQ-9 assessed at baseline and Weeks 4, 8, 12, 16, 20, 24, 36, 48, 52, 60, and 72, plus 4 weeks	Depression (PHQ-9); mental health-related quality of life (SF-36v2 Mental Component Summary and Mental

	post-treatment; SF-36v2 assessed at baseline and Weeks 36 and 72.	Health domain).
