# Supplementary material for: Neuropsychiatric Outcomes Associated with GLP-1 Receptor Agonists in Adults with Overweight or Obesity: A Systematic Review
Source: Brain Sci. 2026 Aug 31;16(9):926. doi: 10.3390/brainsci16090926 (PMC13604221; doi:10.3390/brainsci16090926)
Supplement: Supplementary file 1 [file brainsci-16-00926-s001.zip › Supplementary Table S2.pdf]

Supplementary Table S2. Characteristics of observational cohort studies included in the systematic review

First author, year	Country / Data source	Design	Study objective	Population	Sociodemographic and clinical characteristics	Exposure / Comparator	Confounding control method	Follow-up	Neuropsychiatric outcomes evaluated
**Kuckuck et al., 2026**	The Netherlands; Obesity Center CGG, Erasmus MC, University Medical Center Rotterdam	Observational, one-arm, real-world intervention study	To examine changes in mental health and wellbeing after 4 months of liraglutide 3.0 mg treatment in patients with obesity and to explore associations with weight loss.	Patients with obesity treated with liraglutide 3.0 mg.	N = 98; 72.4% women; median age 39.0 years; median BMI 41.4 kg/m²; 13.5% antidepressant use.	Liraglutide 3.0 mg / baseline (pre-treatment).	Generalized least squares models adjusted for age and sex; multiple linear regression analyses adjusted for age and sex.	4 months	Hospital Anxiety and Depression Scale (HADS): depression and anxiety; OBESI-Q psychological wellbeing.
**Rutledge et al., 2026**	United States; Veterans Affairs San Diego Healthcare System (VA weight management clinic)	Observational, retrospective, one-arm pre–post study	To evaluate changes in psychiatric symptoms, eating disorder behaviors, and health-related quality of life following 6 months of semaglutide or tirzepatide	Military veterans with obesity receiving semaglutide or tirzepatide through a multidisciplinary VA weight management clinic.	N = 90; 63.3% male; mean age 49.7 years; obesity with ≥1 weight-related metabolic condition.	Semaglutide or tirzepatide / within-person baseline comparator (pre-treatment vs 6 months).	None (within-subject paired comparisons using paired t-tests; no multivariable adjustment).	6 months	Depression (BDI-II), suicidal ideation (BDI-II item 9),).

			treatment for obesity.						
**Tang et al., 2025**	United States; TriNetX electronic health records (federated network of healthcare organizations).	Target trial emulation using a retrospective observational cohort.	To compare the effectiveness and safety of GLP-1 receptor agonists versus other anti-obesity medications on cardiovascular, kidney, mental health, and safety outcomes among adults with obesity without diabetes.	Adults (≥18 years) with obesity (BMI ≥30 kg/m² or ICD-10 obesity diagnosis) and no history of diabetes. After 1:1 propensity score matching, 140,169 GLP-1RA users and 140,169 users of other anti-obesity medications were included.	Mean age 48 years; 71% women; 70% White. All participants had obesity without diabetes. Patients with previous diabetes, glucose-lowering therapy, HbA1c ≥6.5%, prior GLP-1RA or anti-obesity medication use, end-stage kidney disease, dialysis, or previous study outcomes were excluded. Baseline characteristics included BMI, comorbidities, medication use, laboratory values, and healthcare utilization.	GLP-1 receptor agonists (liraglutide, semaglutide, or tirzepatide) versus other anti-obesity medications (orlistat, naltrexone/bupropion, phentermine/topiramate, or lorcaserin).	1:1 propensity score matching using multivariable logistic regression adjusting for demographics, BMI, race/ethnicity, comorbidities, medication use, laboratory values, and healthcare utilization. Hazard ratios estimated using Cox proportional hazards models.	Mean follow-up: 392 days for GLP-1RA users and 564 days for comparator users. Follow-up began 90 days after treatment initiation and continued until outcome occurrence, death, last clinical encounter, or end of study.	Depression, suicidal ideation/attempt, and substance use disorder. GLP-1RA use was associated with lower risks of depression (HR 0.63; 95% CI 0.61–0.65), suicidal ideation/attempt (HR 0.42; 95% CI 0.35–0.51), and substance use disorder (HR 0.58; 95% CI 0.55–0.60).

**Kornelius et al., 2024**	United States; TriNetX database (66 healthcare organizations).	Retrospective population-based cohort study.	To investigate the association between GLP-1 receptor agonist therapy and the risk of depression, anxiety, and suicidal behavior among adults with obesity.	Adults (≥18 years) with obesity (BMI ≥30 kg/m² or obesity diagnosis) between 2015 and 2023. After exclusions and 1:1 propensity score matching, 162,253 GLP-1RA users and 162,253 non-users were included.	Mean age 52.4 years; 55.6% female; 61.0% White. Baseline comorbidities included hypertension (51.1%), type 2 diabetes (50.0%), chronic respiratory disease (10.0%), ischemic heart disease (8.7%), chronic kidney disease (6.1%), neoplasms (10.8%), psychoactive substance use (5.2%), and socioeconomic problems related to housing/economic circumstances (0.5%). Patients with bipolar disorder, schizophrenia, depression, anxiety, or suicidal behavior within one year before	GLP-1 receptor agonists (liraglutide: Victoza® 1.8 mg, Saxenda® 3.0 mg; semaglutide: Ozempic® 1 mg, Wegovy® 2.4 mg) versus patients with obesity not receiving GLP-1RA therapy.	1:1 propensity score matching (PSM) adjusting for age, sex, race, socioeconomic factors, and major comorbidities. Hazard ratios estimated using time-to-event analyses.	Up to 5 years (Wegovy subgroup up to 3 years). Psychiatric outcomes assessed at 6 months, 1 year, 3 years, and 5 years.	Any psychiatric disorder, major depressive disorder, anxiety, suicidal ideation, suicide attempts, and intentional self-harm. GLP-1RA use was associated with higher risks of any psychiatric disorder (HR 1.98), major depression (HR 2.95), anxiety (HR 2.08), and suicidal behavior (HR 2.06).

				the index date were excluded.					
**Hurtado et al., 2024**	Spain; Valencia Health System Integrated Database (VID), including linked population-based electronic health records from the Valencia Health System.	Population-based retrospective cohort study (new-user, active-comparator design).	To assess whether initiation of GLP-1 receptor agonists is associated with an increased risk of suicidal ideation and self-injury compared with SGLT-2 inhibitors among adults with type 2 diabetes and obesity.	Adults (≥18 years) with type 2 diabetes and obesity initiating GLP-1RA or SGLT-2i therapy between 2015 and 2021. A total of 14,667 participants were included (3,040 GLP-1RA users and 11,627 SGLT-2i users).	Mean age 59 years; 53.7% men. At baseline, 71.2% had hypertension, 43.1% anxiety, 36.0% sleep disorders, 20.1% depression, and all participants had obesity (BMI ≥30 kg/m² or diagnosis of obesity). After IPTW, baseline covariates were well balanced between groups.	GLP-1 receptor agonists (primarily liraglutide, semaglutide, dulaglutide, exenatide, or lixisenatide) versus SGLT-2 inhibitors used for treatment of type 2 diabetes in patients with obesity.	Inverse probability of treatment weighting (IPTW) based on propensity scores; multivariable Cox proportional hazards regression; new-user active-comparator design; intention-to-treat and per-protocol sensitivity analyses; stratified analyses.	Per-protocol: mean 483 days (GLP-1RA) and 397 days (SGLT-2i). Intention-to-treat: mean 992 days (GLP-1RA) and 970 days (SGLT-2i).	Suicidal ideation and self-injury (SIS), identified from ICD-coded healthcare records. No increase in SIS risk was observed with GLP-1RA versus SGLT-2i.
**Wang et al., 2024**	United States; TriNetX Research US Collaborative Network (electronic health	Retrospective cohort study	To assess the association between semaglutide use and the risk of incident and recurrent	Adults with overweight/obesity or type 2 diabetes prescribed semaglutide or comparator medications. Separate	Overweight/obesity cohort (after propensity score matching): 52,783 patients per group; mean age 50.1 years; 72.6% female. T2DM	Semaglutide vs non-GLP-1 anti-obesity medications (bupropion, naltrexone, orlistat, topiramate, phentermine, setmelanotide) in the	1:1 propensity score matching; Kaplan–Meier survival analysis; Cox	Primary analysis: 6 months. Additional analyses in the T2DM	Incident suicidal ideation; recurrent suicidal ideation; suicide attempts (exploratory,

	records from 59 healthcare organizations).		suicidal ideation compared with non-GLP-1 receptor agonist anti-obesity and anti-diabetes medications.	cohorts were analyzed for overweight/obesity and T2DM.	cohort (after propensity score matching): 27,276 patients per group; mean age 57.5 years; 49.2% female.	overweight/obesity cohort; semaglutide vs non-GLP-1 anti-diabetes medications (insulin, metformin, sulfonylureas, α-glucosidase inhibitors, thiazolidinediones, DPP-4 inhibitors, SGLT2 inhibitors) in the T2DM cohort.	proportional hazards models; subgroup analyses by age, sex, race, and ethnicity.	cohort at 1, 2, and 3 years.	insufficient events for statistical analysis).
**Yu et al., 2025**	United States; TriNetX US Collaborative Network (66 healthcare organizations)	Retrospective cohort study	To evaluate the association between tirzepatide use and the risk of suicidal ideation or suicide attempts compared with other anti-obesity medications in patients with overweight or obesity.	Patients with overweight or obesity prescribed tirzepatide or other anti-obesity medications; patients with prior suicidal ideation, suicide attempts, or previous GLP-1 receptor agonist use were excluded.	After propensity score matching: N = 32,642 (16,321 tirzepatide; 16,321 comparator); mean age 48.4 years; 69.1% female; approximately 29% had type 2 diabetes.	Tirzepatide vs non-GLP-1 anti-obesity medications (bupropion, naltrexone, orlistat, topiramate, phentermine, or setmelanotide).	1:1 propensity score matching (greedy nearest-neighbor matching with a 0.1 caliper); Kaplan–Meier analysis; Cox proportional hazards models; subgroup analyses.	Median 365 days (1 year).	Suicidal ideation; suicide attempts (composite outcome).
**Chang et al., 2026**	Taiwan; TriNetX deidentified electronic	Population-based retrospective cohort	To compare the risk of incident depression	Adults (≥18 years) with newly diagnosed type	N = 51,408 after 1:1 propensity score matching (25,704	Glucagon-like peptide-1 receptor agonists (tirzepatide,	1:1 propensity score matching;	1 year	Incident depression (composite of new

	health record network	study (new-user, active-comparator cohort study)	between glucagon-like peptide-1 receptor agonists and sodium-glucose cotransporter-2 inhibitors in overweight or obese adults with newly diagnosed type 2 diabetes.	2 diabetes and overweight or obesity initiating a glucagon-like peptide-1 receptor agonist or a sodium-glucose cotransporter-2 inhibitor; patients with prior mood disorders or antidepressant use were excluded.	glucagon-like peptide-1 receptor agonist users and 25,704 sodium-glucose cotransporter-2 inhibitor users); mean age 56.8 years; 48.9% male.	semaglutide, or liraglutide) vs sodium-glucose cotransporter-2 inhibitors (canagliflozin, dapagliflozin, or empagliflozin).	Cox proportional hazards models; Kaplan–Meier analysis; time-varying analyses; multiple prespecified sensitivity analyses.		depression diagnosis or antidepressant initiation); depression diagnosis; antidepressant initiation.
**Her et al., 2025**	United States; Merative® MarketScan Commercial Claims and Encounters Databases	Population-based retrospective cohort study (active comparator new-user design)	To evaluate the risk of incident suicidal ideation and suicidality among adults with overweight or obesity prescribed semaglutide for weight management compared with active comparator weight	Adults with overweight or obesity initiating semaglutide or active comparator weight management drugs, without prior suicidality or type 2 diabetes.	N = 28,808 (16,822 semaglutide; 11,986 active comparators); mean age 46.2 and 44.2 years; 77.0% and 81.7% women, respectively.	Semaglutide vs bupropion/naltrexone, orlistat, phentermine, or phentermine/topiramate.	Standardized mortality ratio weighting based on propensity scores.	183 days (6 months).	Suicidal ideation; suicidality.

	management drugs.	
