# Supplementary material for: Neuropsychiatric Outcomes Associated with GLP-1 Receptor Agonists in Adults with Overweight or Obesity: A Systematic Review
Source: Brain Sci. 2026 Aug 31;16(9):926. doi: 10.3390/brainsci16090926 (PMC13604221; doi:10.3390/brainsci16090926)
Supplement: Supplementary file 1 [file brainsci-16-00926-s001.zip › Supplementary Table S3.pdf]

Supplementary Table S3. Characteristics of the cross-sectional study included in the systematic review

First author, year	Country / Data source	Design	Study objective	Population	Sociodemographic and clinical characteristics	Exposure	Neuropsychiatric outcomes evaluated
**Witaszek et al., 2024**	Poland; online survey (CAWI)	Cross-sectional analytical study	To evaluate the association between symptoms of depression and anxiety and anti-obesity treatment among adult women across different BMI categories.	Adult women (≥18 years) residing in Poland, across normal weight, overweight, and obesity BMI categories.	N = 1,105; mean age 38.89 ± 9.00 years; mean BMI 33.32 ± 6.67 kg/m²; 23.0% overweight and 68.7% obesity; 44.2% used anti-obesity medication.	Anti-obesity pharmacotherapy: semaglutide (n = 232), liraglutide (n = 235), and naltrexone/bupropion (n = 41); previous bariatric surgery was also evaluated.	Depressive symptoms (PHQ-9) and generalized anxiety symptoms (GAD-7).
