# Supplementary material for: Neuropsychiatric Outcomes Associated with GLP-1 Receptor Agonists in Adults with Overweight or Obesity: A Systematic Review
Source: Brain Sci. 2026 Aug 31;16(9):926. doi: 10.3390/brainsci16090926 (PMC13604221; doi:10.3390/brainsci16090926)
Supplement: Supplementary file 1 [file brainsci-16-00926-s001.zip › Supplementary Table S5.pdf]

Supplementary Table S5. Complete electronic search strategies used for the systematic review.

Database	Search strategy	Date searched
PubMed	(("glucagon-like peptide 1 receptor"[MeSH] OR "glucagon-like peptide-1 receptor agonist*"[tiab] OR GLP-1[tiab] OR semaglutide[tiab] OR liraglutide[tiab] OR dulaglutide[tiab] OR exenatide[tiab] OR lixisenatide[tiab] OR tirzepatide[tiab]) AND (depress*[tiab] OR anxiety[tiab] OR suicid*[tiab] OR self-harm[tiab] OR self-injur*[tiab] OR psychiatr*[tiab] OR "mental health"[tiab] OR mood[tiab] OR cognit*[tiab] OR mania[tiab] OR manic[tiab] OR hypomania[tiab] OR hypomanic[tiab] OR bipolar[tiab] OR "Mental Disorders"[MeSH] OR "Suicidal Ideation"[MeSH] OR "Bipolar Disorder"[MeSH]) AND (obesity[MeSH] OR obes*[tiab] OR overweight[tiab] OR "weight loss"[tiab] OR "weight management"[tiab]))	13 June 2026
Scopus	("adipose tissue hyperplasia" OR adipositas OR adiposity OR corpulency OR obesitas OR obesity OR "body weight, excess" OR "excess body weight" OR overweight) AND ("glp 1 agonist" OR "glp 1 receptor agonist" OR "glucagon like peptide 1 agonist" OR "glucagon like peptide 1 receptor agonist" OR "glucagon like peptide 1 receptor stimulating agent" OR "glucagon-like peptide-1 receptor agonists" OR "incretin mimetics" OR "long acting glp 1 agonist" OR "long acting glp 1 receptor agonist" OR "long acting glucagon like peptide 1 agonist" OR "long acting glucagon like peptide 1 receptor agonist" OR semaglutide OR liraglutide OR dulaglutide OR exenatide OR lixisenatide OR tirzepatide) AND ("central depression" OR "clinical depression" OR depression OR "depressive disease" OR "depressive disorder" OR "depressive episode" OR "depressive illness" OR "depressive personality disorder" OR "depressive state" OR "depressive symptom" OR "depressive syndrome" OR depressivity OR "mental depression" OR "parental depression" OR "anxiety disorder" OR "anxiety disorders" OR mania OR hypomania OR "manic behavior" OR "manic behaviour" OR "manic disorder" OR "manic episode" OR "completed suicide" OR "self killing" OR "suicidal completion" OR "suicidal death" OR "suicidal fatality" OR "suicidal poisoning" OR suicide OR "suicide death" OR "suicide, completed" OR suicidium OR suicidum OR "abnormal mental state" OR "abnormal mental state" OR "disease, mental" OR "diseased mental state" OR "disorder, mental" OR "disordered mental state" OR "disturbed mental state" OR "illness, mental" OR insanity OR "mental abnormality" OR "mental change" OR "mental confusion" OR "mental defect" OR "mental disease" OR "mental disorder" OR "mental disorders" OR "mental disorders diagnosed in childhood" OR "mental disturbance" OR "mental health condition" OR "mental health disease" OR "mental health disorder" OR "mental health issue" OR "mental health problem" OR "mental illness" OR "mental insufficiency" OR "mental symptom" OR "mentally ill" OR "neurodevelopmental disorder" OR "neurodevelopmental disorders" OR	13 June 2026

Database	Search strategy	Date searched
	"neuropsychiatric disease" OR "neuropsychiatric diseases" OR "neuropsychiatric disorder" OR "neuropsychiatric disorders" OR "psychiatric disease" OR "psychiatric disorder" OR "psychiatric illness" OR "psychiatric symptom" OR "psychic disease" OR "psychic disorder" OR "psychic disturbance" OR "psychologic disorder" OR "psychologic disturbance" OR "psychological disorder" OR "psychological disturbance" OR psychopathology)	
Web of Science	(("glp-1 receptor agonist*" OR "glucagon-like peptide-1 receptor agonist*" OR semaglutide OR liraglutide OR dulaglutide OR exenatide OR lixisenatide OR tirzepatide) AND (depress* OR anxiety OR suicid* OR mania OR hypomania OR manic OR bipolar OR psychiatr* OR "mental health" OR psychopathology) AND (obes* OR obesity OR overweight OR adiposity))	13 June 2026

Abbreviations: MeSH, Medical Subject Headings; tiab, title/abstract.

Note: Searches were restricted to records published within the preceding 10 years. Full-text eligibility was limited to articles published in English or Spanish. Search strategies were adapted to the syntax requirements of each database while maintaining the same conceptual framework.
